# Potential of 3D printing technologies for fabrication of electron bolus and proton compensators

**DOI:** 10.1120/jacmp.v16i3.4959

**Published:** 2015-05-08

**Authors:** Wei Zou, Ted Fisher, Miao Zhang, Leonard Kim, Ting Chen, Venkat Narra, Beth Swann, Rachana Singh, Richard Siderit, Lingshu Yin, Boon‐keng Kevin Teo, Michael Mckenna, James McDonough, Yue J. Ning

**Affiliations:** ^1^ Department of Radiation Oncology Rutgers Cancer Institute of New Jersey New Brunswick NJ USA; ^2^ Department of Radiation Oncology Community Cancer Care Missoula MT USA; ^3^ Department of Radiation Oncology University of Pennsylvania Philadelphia PA USA; ^4^ Department of Pathology Rutgers Cancer Institute of New Jersey New Brunswick NJ USA

**Keywords:** electron bolus, proton compensator, 3D printing

## Abstract

In electron and proton radiotherapy, applications of patient‐specific electron bolus or proton compensators during radiation treatments are often necessary to accommodate patient body surface irregularities, tissue inhomogeneity, and variations in PTV depths to achieve desired dose distributions. Emerging 3D printing technologies provide alternative fabrication methods for these bolus and compensators. This study investigated the potential of utilizing 3D printing technologies for the fabrication of the electron bolus and proton compensators. Two printing technologies, fused deposition modeling (FDM) and selective laser sintering (SLS), and two printing materials, PLA and polyamide, were investigated. Samples were printed and characterized with CT scan and under electron and proton beams. In addition, a software package was developed to convert electron bolus and proton compensator designs to printable Standard Tessellation Language file format. A phantom scalp electron bolus was printed with FDM technology with PLA material. The HU of the printed electron bolus was 106.5±15.2. A prostate patient proton compensator was printed with SLS technology and polyamide material with −70.1±8.1 HU. The profiles of the electron bolus and proton compensator were compared with the original designs. The average over all the CT slices of the largest Euclidean distance between the design and the fabricated bolus on each CT slice was found to be 0.84±0.45 mm and for the compensator to be 0.40±0.42 mm. It is recommended that the properties of specific 3D printed objects are understood before being applied to radiotherapy treatments.

PACS number: 81.40

## I. INTRODUCTION

During radiation therapy treatments, compensators or bolus are often used to achieve desired dose distribution. In electron therapy, customized bolus is particularly useful for the treatment of shallow tumors^1^, ^2^, ^3^, ^4^, ^5^ at various sites. The patient‐specific bolus reduces irradiation to healthy tissues and increases dose homogeneity for patients with complex surface contours and varying target depths.^3^, ^6^, ^7^ The bolus is usually made of water‐equivalent material, such as wax, and applied directly over patient skin surface. Patient‐specific electron bolus design algorithms have been developed.^8^, ^9^ Vyas et al.^(10)^ provided a comprehensive review and guidance of various bolus materials used in electron radiation therapy.

In proton radiotherapy, a series of monoenergetic proton beams are delivered to form a spread‐out Bragg peak (SOBP) to provide adequate target coverage in the treatment beam direction.^(11)^ With the proton double scattering treatment technique, patient‐specific compensator is a necessary beam modifier to shape the dose distribution to the distal end of the tumor target.^(12)^ The proton compensator is designed in the treatment planning system, and can be fabricated out of paraffin wax or acrylic material. The compensator is usually mounted on the treatment nozzle and aligned with beam axis. To achieve desired precision, the electron bolus and proton compensator has been traditionally fabricated on a milling machine. However, a large amount of the material is milled out during the milling process and gets wasted. The quality assurance of the electron bolus usually involves checking the electron dose distribution based on the fabricated bolus and the patient CT images. Several literatures presented the proton compensator QA from the CT scan of the fabricated compensators to verify its geometry against the designs.^13^, ^14^, ^15^

Three‐dimensional (3D) printing technologies are becoming popular that can be used to build complex volumetric objects in virtually any shape.^(16)^ The technologies print designed 3D objects through successive very fine layers. The resolution of the printed objects can be less than 100 μm.^(17)^ Various materials including plastic, ceramics, and metals can be used for printing. 3D printing is in fact a general term for several printing technologies. These technologies differ in their processes of the 3D layer deposition and the printing materials. Popular technologies include fused deposition modeling (FDM) and selective laser sintering (SLS). The fine resolution and the capability of printing objects of virtually any shapes make the 3D printing technologies suitable for the fabrication of bolus and compensators in electron and proton radiotherapy. The 3D printing technologies have since been utilized in radiotherapy. Kim et al.^(18)^ 3D‐printed a mouse stereotactic body mold using SLA technology for CyberKnife QA studies. Nie et al.^(19)^ 3D‐printed ^192^Ir‐based small animal apparatus. Cundra et al.^(20)^ printed a customized brachy applicator. Fisher et al.^(21)^ pioneered the clinical flow of the patient skin lesion treatment with the 3D‐printed bolus from patient face profiles acquired with a Kinect camera. Su et al.^(9)^ printed from MakerBot printer the electron bolus with their developed design algorithm. Ju et al.^(15)^ evaluated 3D‐printed proton compensator using stereolithography (SLA) 3D printing technology. The material used in this study was ultraviolet curable acrylic plastic. Other proton radiotherapy applications include 3D printed range spreading filter^(22)^ and patient‐specific bolus for proton.^(23)^ Our study focused on the fabrication process and characterizations of 3D‐printed patient electron bolus and proton compensator using FDM and SLS technologies with PLA and polyamide materials, respectively. The printed materials were characterized for their printed qualities with CT scans and dosimetric effects under the electron and proton beams. The fabricated electron bolus and proton compensator were also characterized for their printed qualities and physical dimension accuracies.

## II. MATERIALS AND METHODS

### A. Material characterization

To assess the 3D printing materials used for electron and proton radiotherapy, 3D‐printed cubes were prepared with FDM and SLS 3D printing technologies. Two $3\times 3\times 3\;{\text{cm}}^{3}$ cubes were printed with the FDM technology with PLA material on a consumer model MakerBot Replicator II printer (MakerBot Industries LLC, Brooklyn, NY). Two $4\times 4\times 4\;{\text{cm}}^{3}$ cubes were printed with SLS technology with polyamide PA2200 material on a commercial EOS 3D printer (EOS, Krailling, Germany). These samples were CT‐imaged on a GE LightSpeed 16 CT scanner (GE Healthcare, Waukesha, WI) at 0.625 mm slice spacing. The region within the cube that was 1.25 mm from the air/cube interfaces was used to examine their Housefield units (HUs) and internal uniformity.

**Figure 1:**
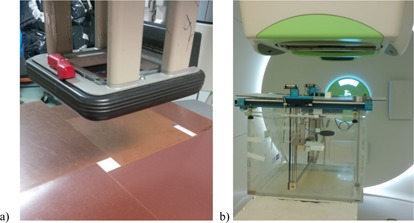
Characterization setup for 3D printed cubes under (a) electron beam with a field projection of 10×10 cm cone at 100 cm SSD, and (b) proton monoenergetic pencil beam.

The materials were further characterized under electron and proton beams. Under electron beams with $10\times 10\;{\text{cm}}^{2}$ cone, the samples were placed at 100 cm SSD surrounded by Solid Water phantom (Fig. 1(a)). In the setup, one horizontal and one vertical Gafchromic EBT2 films (ISP, Wayne, NJ) were inserted underneath the cube sample to collect the dose distributions. The vertical film was aligned with the midline of the $10\times 10\;{\text{cm}}^{2}$ field. Such setup was rebuilt in Eclipse treatment planning system (Varian Medical Systems, Palo Alto, CA) and the dose distributions calculated with eMC algorithm with 0.2 cm grid size. The dose distributions at the film planes were output to compare with the film measurements.

To assess the proton stopping power, these samples were placed on an 8 mm thick Plexiglass board above a water tank (Fig. 1(b)). A monoenergetic proton pencil beam with 15.0 cm range was used to irradiate the sample. The depth‐dose curve change was collected using Bragg peak ionization chambers (PTW, Freiburg, Germany). GEANT4 Monte Carlo simulation was also performed to compare the depth‐dose curve with the experiment. In the Monte Carlo simulation, the experimental setup was structured in the model. The 3D‐printed material composition and their measured density was built‐in. The dose was collected in a series of scorers the size of the Bragg peak chamber along the water depth. The measured depth‐dose distributions were then compared with the GEANT4 Monte Carlo simulations.

### B. 3D‐printed bolus and compensator fabrication

3D‐printed bolus and compensator design were performed based on patient CT images that were acquired for treatment planning purpose. The CT HU corresponds to the electron density or the proton stopping power. The contours of patient external body surface, planned treatment volume (PTV), and organs at risk (OARs) were drawn on the CT images. Incorporating the material electron density, the electron bolus can be designed with the procedures provided by Low et al.^(8)^ and Su et al.^(9)^ or with commercial software, such as p.d software (.decimal, Sanford, FL). In this study, an electron bolus was designed for contoured PTV in an anthropomorphic head phantom. The design was performed in p.d software with the input of the material property measured from the section above. The resulting bolus design was embedded in the DICOM structure file in the Eclipse treatment planning system. Dose calculation was carried out to ensure adequate dose coverage to the PTV. The proton compensator was designed for a prostate patient receiving two lateral proton beams. The stopping power ratio of the 3D printing material was input into the Eclipse beam configuration. Based on the proton ray tracing algorithm and proton stopping power ratio to water of the tissues and the printing material, the compensator design was performed by TPS to provide sufficient CTV/PTV coverage. The compensator profile was embedded in the Eclipse proton plan DICOM file.

The bolus and compensator design, as saved in the digital DICOM files, consisted of a series of points with their corresponding Cartesian coordinates. An in‐house program written in MATLAB was developed to generate a mesh to interconnect these points to define the surfaces of the bolus and compensator. To increase the resolution of the printed part, interpolation was applied to the surface profiles to be less than 1 mm before meshing. The contours of the electron bolus structure had fine resolutions usually <1 mm on each CT axial slice. Therefore, the interpolation was performed between the CT slices to reduce the CT slice spacing to be 1 mm. Different from electron bolus, digitally exported proton compensator design from Eclipse TPS was in 1 mm resolution; therefore, no additional interpolation was performed.

To generate the mesh interconnecting the surface points, the Delaunay triangulation was selected for meshing reconstruction.^(24)^ The triangulation process sorts through the bolus and compensator surface point clouds and builds triangles with the provided points in such a way that no residual point exists in the circumcircle of any triangulation. The built‐in Delaunay triangulation in MATLAB software was selected for this task. After meshing, the vertices and normals of the triangles were written into the Standard Tessellation Language (STL) file format^(25)^ which most commercial 3D printers accept and can generate machine code (g code) for printing. The file was then transferred to a 3D printer for printing.

In this study, the electron bolus was designed and printed with PLA material from the MakerBot ReplicatorII 3D printer with FDM technology. The proton compensator was printed in polyamide material PA 2200 with laser sintering SLS technology on the EOS 3D printer. The compensator printing dimension was scaled by 0.5 in each direction from the design to save printing time. The printed objects were printed with 100% solid fill inside.

### C. 3D‐printed bolus and compensator characterization

The dimensions of the printed electron bolus and proton compensators were measured and compared with the design model. The overall dimensions were measured with a caliper. For proton compensator, a Mitutoyo QA‐height gauge (Mitutoyo Corp., Aurona, IL) was employed to measure several known points at relatively flat regions from the design. The measured heights from the bottom of the compensator were compared with the design. Such measurement was not performed for the electron bolus as there were no identifiable flat regions in the design.

In addition, the electron bolus and proton compensator were then CT scanned. The surface profile of the printed objects were derived from CT images and compared with the design. The Euclidean distance was calculated between the profile points between the physical printed bolus/compensator and the design. The largest Euclidean distance was recorded as an index of the conformity of the printed bolus/compensator profiles with the design. The dose distribution on the anthropomorphic phantom was also calculated with the printed electron bolus in place to examine the dose coverage.

## III. RESULTS

### A. Material characterization

The average and standard deviation HU of the PLA cubes were determined to be 130.1±10.1, and for the polyamide cubes −72.1±5.3. The density of the printed PLA cube and polyamide cubes were measured to be $1.19\pm 0.03\;g/{cm}^{3}$ and $0.97\pm 0.02\;g/{cm}^{3}$, respectively.

**Figure 2:**
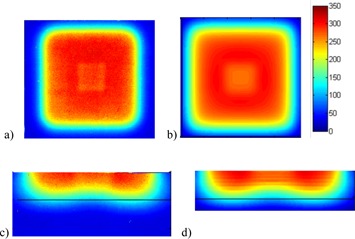
Comparison of the film measurements with the TPS dose calculations for the PLA cube irradiated with 300 MU 12 MeV electron beam. The horizontal planar dose distribution at 3 cm depth underneath the cube (a) on the film and (b) TPS calculation. The vertical dose distribution beyond 3 cm depth under the cube (c) on a film and (d) from TPS calculation. The black line in (c) and (d) marks the 5 cm depth. The color bar shows dose in cGy.

With the setup in Fig. 1(a), 300 MU electron beams with various energies were delivered. The films were then scanned and converted to the dose distribution with the OD vs. dose calibration curve for the same film batch. Figure 2 shows the comparison of the film measurements with the TPS dose calculations for the PLA cube irradiated with electron beam under 10×10 cm cone. Due to the higher density of the PLA material compared to water, lower dose regions were observed underneath the cube. The depth dose distribution also showed the larger attenuation effect of the PLA cube compared to water. Good dose agreements were observed for both the horizontal and the vertical planar dose distributions.

**Figure 3:**
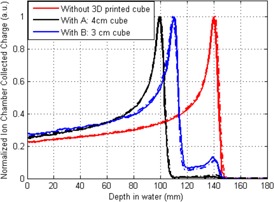
Proton depth0dose curves under monoenergetic pencil beam with and without the 3D printed cubes intercepting the beam. Sample A was the 4 cm polyamide cube, B was the 3 cm PLA cube. Solid lines correspond to measured depth‐dose curves and dashed lines correspond to Monte Carlo‐simulated curves.

Figure 3 shows the relative proton dose in water collected by the PTW Bragg peak chamber with and without the printed cubes intercepting the proton pencil beam. The measured dose curves were in agreement with the GEANT4 Monte Carlo simulated curves. The depth curves were also measured with different orientation direction of the printed cube relative to the proton beam axis. The depth‐dose shift due to the cube orientation was undetectable. This result showed that the proton beam is insensitive to the 3D printing directions. The pull‐back of the Bragg peak on the depth‐dose curves can be used to calculate the proton stopping power relative to water. The obtained average proton stopping power relative to water was 1.10 for printed PLA material and was 0.98 for printed polyamide material.

### B. 3D‐printed bolus and compensator fabrication

**Figure 4:**
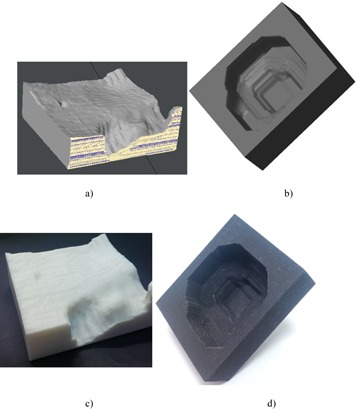
3D rendering of (a) electron bolus and (b) proton compensator from the model STL files. The printed (c) electron bolus (d) proton compensator.

An electron bolus was designed from .decimal software with the correct input of the PLA material for an anthropomorphic head phantom. The bolus structure interpolation and meshing were performed, as described in Materials & Methods section B. The generated STL file following the meshing process was examined with the CAD software netfabb (netfabb GmbH, Parsberg, Germany) for the integrity of the STL model. Figure 4(a) shows the 3D rendering of the designed electron bolus. This PLA electron bolus was printed from a MakerBot Replicator II printer, as shown in Fig. 4(c).

The proton compensator was designed with the input of the proton stopping power obtained from previous section. The compensator had a concaved surface to shape the dose distribution to the distal end of the prostate target volume. The design was then meshed and the printable STL file was generated. Figure 4(b) shows the 3D rending of the proton compensator. This proton compensator was printed with polyamide from an EOS printer, as shown in Fig. 4(d).

### C. 3D‐printed bolus and compensator characterization

The electron bolus and proton compensator were first CT scanned to examine the uniformity in the printing processes. The internal HU for the printed electron bolus was shown as 106.5±15.2. The mean HU differs from the printed PLA cubes by about 14 HU with larger standard deviation. The mean HU from the printed polyamide proton compensator was determined −70.1±8.1, very close to the HU from the printed cube. The mass density was estimated from the bolus and compensator weight divided by the design volumes. This commercial EOS SLS technology shows more consistent printing qualities compared with the MakerBot II FDM technology. Nine identifiable flat regions on the proton compensator were characterized with a Mitutoyo QA‐height gauge to compare with the design. The measured deviations were less than 1.0 mm with the uncertainties of the whereabouts of measurement points taken into account.

**Figure 5:**
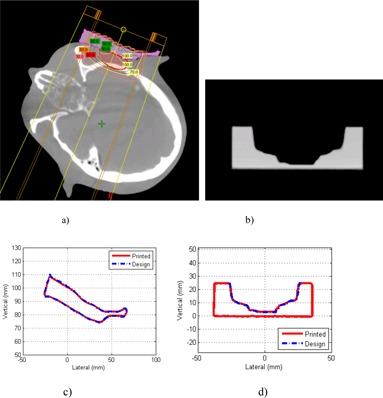
Example of a CT slice of the 3D‐printed (a) electron bolus, (b) proton compensator; and the scanned profiles of (c) electron bolus and (d) proton compensator in comparison with the design.

To examine the entire bolus profiles, the bolus and compensator profiles were extracted from the scanned CT images. The profiles were compared with the design profile and are shown in Fig. 5. The average over the CT slices of the largest Euclidean distance between the design and the fabricated bolus was found to be 0.84±0.45 mm and between the design and fabricated compensator 0.40±0.42 mm.

## IV. DISCUSSION

In most of current practice, the electron bolus and proton compensators are fabricated on milling machines where a drilling bit drills out a pattern on a solid piece of material. The pattern can be limited by the stepping and the drilling bit size. Different from milling machines, the 3D printing technologies print layer by layer with fine resolution and can be used to print much finer patterns on the compensator. It can also be used to print certain irregularly shaped patterns that cannot be fabricated with a milling machine due to the blocked access of the drilling bit to certain locations. Compared to the traditional milling machine technologies, 3D printing can realize more complex bolus and compensator designs and provide potentially more flexibility in developing patient treatment plans. This is a great advantage of using 3D printing technologies for bolus and compensator fabrications.

The electron bolus and the PLA cubes were printed with solid infill with the FDM 3D printing technology on a MakerBot Replicator II printer. This technology involves melting a fine strand of PLA material during extrusion and depositing it at desired spatial position. During the extrusion and curing of the PLA material, slight inhomogeneity happens which could be due to the heat dissipation during the deposition. Variations were observed among samples, evidenced by the measured HU variations between the printed cubes to the printed electron bolus. The standard deviations of the HUs within these samples were also larger than the samples printed with polyamide material (Table 1). In TPS calculation, the variations in HU create less than 1 mm change in the depth of 90% isodose line of electron beam. Such deviations in depth‐dose coverage are usually acceptable in electron radiotherapy. Therefore, we recommend good knowledge of the printed subjects in terms of its homogeneity before applying to electron radiotherapy.

**Table 1:** List of measured CT HUs and corresponding material properties.

	*Samples*	*HUs*	*Mass Density* $\left(g/{\text{cm}}^{3}\right)$	*Electron Density Relative to Water*	*Proton Stopping Power Relative to Water*
1	Electron bolus with PLA	106.5±15.2	1.19±0.01	1.068±0.004	1.093±0.007
2	$3\;{\text{cm}}^{3}$ PLA cube	130.1±10.1	1.19±0.03	1.073±0.002	1.102±0.004
3	Proton compensator with polyamide	−70.1±8.1	0.97±0.02	0.940±0.006	0.979±0.009
4	$4\;{\text{cm}}^{3}$ polyamide cube	−72.1±5.3	0.97±0.02	0.939±0.004	0.977±0.006

Due to the significant dose effect from material heterogeneity in proton radiotherapy,^(26)^ cautions should be taken when applying the FDM printing technology with the MakerBot II printer. Thorough dosimetric evaluation on the printing variations is suggested before applying this technology to proton radiotherapy. The SLS printing technology produced more uniform polyamide proton compensator. Very small variations in HUs were observed within and among the samples. This printing technology should be suitable for the fabrication of the proton compensators.

3D printing technologies provide an affordable alternative process for fabricating electron bolus and proton compensator. Although the cost of the printing materials is usually low, the price of the printer and the quality of the printed objects combined should be taken into consideration. 3D printing technology can also have size limitation. For example, the consumer model MakerBot II printers have size limit to $28.5\times 15.3\times 15.5\;{\text{cm}}^{3}$ and might not be suitable for printing large bolus, such as chest wall bolus. The other concern is its long printing time compared with milling. The electron bolus and proton compensator in this study took 6 to 9 hrs to print. And the printing time and cost increase with the printing size. Although the printing process needs little personnel attention, such long printing time should be taken into consideration during patient treatment planning and scheduling.

## V. CONCLUSIONS

This paper presents an alternative fabrication process for radiotherapy electron bolus and proton compensators with 3D printing FDM and SLS technologies. It is demonstrated and confirmed that the electron bolus and proton compensators fabricated with appropriate 3D printers can be used in radiotherapy without introducing significant dose deviations. Both technologies can produce fairly uniform geometry to be used in electron and proton therapy. However, due to the random slight inhomogeneity in the FDM printing, the properties of the printed object should be understood before being applied to proton therapy.

## ACKNOWLEDGMENTS

The authors would like to acknowledge .decimal (www.dotdecimal.com) for their technical support in incorporating the 3D printing materials for electron bolus design in the p.d software.
